# Disturbed Monitoring and Response Inhibition in patients with Gilles De La Tourette Syndrome and Co-Morbid Obsessive Compulsive Disorder

**DOI:** 10.1155/2003/832906

**Published:** 2003-01-01

**Authors:** Sandra Verena Müller, Sönke Johannes, Berdieke Wieringa, Axel Weber, Kirsten Müller-Vahl, Mike Matzke, Hans Kolbe, Reinhard Dengler, Thomas F. Münte

**Affiliations:** ^1^Department of Clinical NeuropsychologyOtto-von-Guericke University MagdeburgGermany; ^2^Department of NeurologyHannover Medical SchoolGermany; ^3^Department of Clinical PsychiatryHannover Medical SchoolGermany; ^4^Department of NeurologyOtto-von-Guericke-University of MagdeburgGermany

**Keywords:** Gilles de la Tourette Syndrome, obsessive compulsive disorder neuropsychology, executive functions, response inhibition, monitoring

## Abstract

*Objective:* Fronto-striatal dysfunction has been discussed as underlying symptoms of Tourette syndrome (TS) with co-morbid Obsessive Compulsive Disorder (OCD). This suggests possible impairments of executive functions in this disorder, which were therefore targeted in the present study.

*Results:* A comprehensive series of neuropsychological tests examining attention, memory and executive functions was performed in a group of 14 TS/OCD in co-occurrence with OCD patients and a matched control group.

*Results:* While attentional and memory mechanisms were not altered, TS/OCS patients showed deficits in executive functions predominately in the areas of response inhibition and action monitoring.

*Conclusions:* These findings provide further evidence for a substantial impairment of the frontal-striatal-thalamic-frontal circuit. We propose that the deficits in monitoring, error detection and response inhibition constitute the major impairment of TS/OCD patients in the cognitive domain.

## 1. Introduction

The Gilles de la Tourette Syndrome (TS) is a complex neuropsychiatric disorder, which is characterized by the presence of multiple fluctuating motor and vocal tics [39]. In his first description of the syndrome, Gilles de la Tourette (1885) [13] himself had included obsessive compulsive thinking as part of the disease spectrum. This co-occurrence of Obsessive Compulsive Disorder (OCD) and TS continues to be under study up to the present day with an additional coincidence observed for TS and Attention-Deficit-Hyperactivity-Disorder (ADHD). A recent study from Israel, however, investigating the prevalence of TS in 16–17 year old subjects revealed OCD in 40% of the subjects diagnosed to have TS while only 8% of the TS patients had ADHD, a number not significantly different from the general population [4], thus suggesting a link between TS and OCD but not ADHD. TS and OCD are associated with basal ganglia pathology [30] and a response to dopamine receptor agonists.

ISSN 0953-4180/03/$8.00 © 2003 – IOS Press. All rights reserved

For several patient groups including OCD and TS a hyperactivity of basal ganglia-thalamic frontal cortical loops has been proposed as underlying the neuropsychological deficits in these disorders [29]. With regard to TS and OCD, it has been proposed that dysfunction of basal ganglia-thalamic frontal cortical loops produce "positive" symptoms of excessive activity. This system is part of the neuronal network underlying the so-called executive functions: adaptive behaviour in environmental situations, initiating, execution, release or withholding of various types of response.

A growing number of studies have addressed the neuropsychological profile of OCD [2,28]. No unitary neuropsychological profile of OCD emerged and Alarcón [2] in particular stressed the possible etiological heterogeneity: While some authors described visuospatial memory deficits with no intellectual, attentional or "frontal" deficits [28,42], others observed a frontal lobe dysfunction [20,24]. With regard to OCD, it is proposed that dysfunction of basal ganglia-thalamic frontal cortical loops produce "positive" symptoms of excessive grooming, checking, and doubt most common in OCD [9]. In this respect, doubt probably reflects excessive reliance on external as opposed to internal information for the closure of a cognitive event. Therefore doubt represents a sign of deficient executive control/action monitoring.

It has been stressed that patients with TS or OCD have a disturbed fronto-thalamo-striatal network and (therefore) a diminished ability to handle fast changing situations [24]. More specifically, previous studies have demonstrated impaired action monitoring in OCD [16] and TS patients [21] using a component of the event-related-brain potential, the error related negativity (ERN), which is known to reflect the monitoring of performance errors. However, there has been little systematic investigation of the relative components of executive function amongst adults with Tourette Syndrome [23,37].

No neuropsychological data exist to date about different components of executive function in TS. To adapt their behaviour to an ever-changing environment, humans need to be able to monitor their performance and to detect and correct any errors. This ability is a core function of the executive system. The structure of executive functions is a matter of ongoing investigations and some controversy. Most researchers concur that executive processes are mediated by the prefrontal cortex and are involved in the regulation of processes operating on the contents of working memory [40]. Moreover there is a lack of consensus about the taxonomy of executive processes. Recently executive functions were subdivided at different levels [25,40]. Here we follow a simplified subdivision of executive functions into three subcomponents: (a) monitoring and response inhibition, (b) cognitive productivity and fluency, and (c) task management and planning [26].

Neuropsychological functioning continues to be an important component in understanding the full neurobehavioral spectrum of TS with co-morbid OCD [9]. Keeping in mind the proposed dysfunction of basal-ganglia-thalamic-frontal circuit [30] we hypothesize disturbed executive functions, especially disturbed monitoring and response inhibition. To gain a more comprehensive view of the neuropsychological profile especially about the different subcomponents of the executive functions in TS patients in combination with OCD we conducted a series of neuropsychological tests with adult TS/OCD patients and a matched control group focussing on different types of executive functions. TS is a developmental disorder, the expression of which changes significantly with age, improve often during adulthood. Most of the previous studies [29, 36,38,39] of the cognitive deficits associated with TS have chosen children as participants. It is assumed that group differences would be less pronounced than those in children. Therefore, we decided to investigate an adult sample. Keeping in mind the proposed dysfunction of basal ganglia- thalamic frontal cortical loops [30] in TS/OCD we hypothesise disturbed executive functions, focussing the subcomponent monitoring and response inhibition. Because TS and OCD are closely related and both associated with basal ganglia pathology [30], we decided to investigate a TS patient sample with additional coincidence of OCD.

## 2. Patients and methods

Fourteen native German-speaking adults were diagnosed to fulfil the DSM IV [3] criteria for the diagnosis of TS. The mean age of the patients was 29.2 years (SD = 12.5) and the mean duration of formal education was years 12.4 years (SD = 2.8)[1] (see Table 1). In addition, all patients fulfilled the DSM IV criteria for OCD. In three patients the diagnosis of ADHD was warranted. In all patients TS was verified by two neurologists and

[1] In Germany a three branched education system is used with people attending the Hauptschule ("main school") receiving 9 years of schooling. Usually, the Hauptschule is followed by an apprenticeship in some skilled profession.

one psychiatrist using structured psychiatric interviews, and included only in the case of an agreement among all three clinicians. The patients were free of additional neurological or psychiatric disorders.

The control group was free of neurological or psychiatric disorders. No significant differences between the groups were revealed by t-test with respect to age or years of formal education (see Table 1). The study had been approved by the local institutional review board and informed consent was obtained from all participants.

## 3. Neuropsychological examination

All patients were subjected to an extensive neuropsychological test-battery. Tests on executive functions were classified according to the different subcomponents of executive functions.

### *3.1. Executive functions: Monitoring and response inhibition*

#### *3.1.1. Colour-Word-Interference Test* (*CWIT*) *[17]*

This test measures the capability with which a patient can shift his perceptual set to conform to changing demands. In this version, three cards are presented to the subjects, each containing ten rows of four items. Card A displays coloured dots (four colours), four German words are printed in the colours blue, green, red, and yellow on card B and card C contains colour names printed in some colour other than the colour represented by the word. Card A, B and C are presented in succession with the instruction to name the actual colour of the dot or word. As dependent variables speed and errors of card C were scored.

#### *3.1.2. Response Inhibition* (*Subtest of the Computerized battery for the assessment of attention deficits* (*CBAA*)) *[43]*

Response inhibition is assessed with a go/nogo-task. In this task 40 stimuli, consisting of 20 oblique and 20 upright crosses, are successively presented in random order in the middle of the computer screen. Subjects had to react by pushing a button whenever an upright cross appeared, but are not supposed to react to the oblique stimulus. In this go/nogo paradigm the dependent variables are RTs, errors and omissions.

#### *3.1.3. Response flexibility* (*Subtest of the CBAA*) *[43]*

This experiment tests the ability to engage and disengage the attentional focus. On each trial a letter and a number are presented at the same time, equidistant from the centre on the left and right halves of the screen. Between trials the position of the letter and number varies randomly. A response button corresponds to each half of the screen. In the first condition the letter is the target stimulus. Subjects are instructed to react as fast as possible by pressing the button on the side where the letter appeared. In the second condition the number is the target stimulus and in the third condition subjects had to alternate between numbers and letters. The third condition, which we focus on, requires the capacity to change the focus of attention flexibly without further external instructions. The dependent variables are errors and RTs.

#### *3.1.4. Intermodal comparison* (*Subtest of the CBAA*) *[43]*

This test assessed the ability to integrate visual and auditory information. Targets are defined as an upward pointing arrow followed by a high-pitched tone or a downward pointing arrow followed by a low-pitched tone. 36 target stimuli are randomly interspersed in a series of 80 non-target stimuli. In this go/nogo paradigm RTs, errors and omissions were scored.

### *3.2. Executive functions: Cognitive productivity and fluency*

#### *3.2.1. Controlled oral word association test* (*COWAT*) *[5]*

This test examines access to semantic information (verbal fluency) under time constraints. Patients are instructed to name as many words (excluding names) beginning with the letter F (trials 2 and 3: letters A, S) as they can within one minute. The score is the number of words produced in one minute and perseverative errors. Frontal lesions, left more than right have been found to result in impaired word production.

#### *3.2.2. Ruff figural fluency test* (*RFFT*) *[33]*

This test targets aspects of supervisory attentional control and non-verbal fluency. Materials comprise five sheets of paper, each containing 40 squares. The first sheet consists of squares each containing five symmetrically and identically arranged dots. Of the other four sheets, II and III retain dots in the same position but contain interference patterns; the dots on trials IV and V are asymmetrically positioned with all squares alike

Table 1
Patient characteristics. Data for matched controls shown in brackets. * refers to subjects who were still in education

| Subject | Age | Sex | Handedness | Educational years | Drug Treatment (Pimozide, mg/d) |
|---|---|---|---|---|---|
| 1 | 30 (31) | m (m) | r (r) | 9 (10) | 4 |
| 2 | 34 (32) | f (f) | r (r) | 12 (13) | 6 |
| 3 | 19 (21) | m (m) | r (r) | 11 (10) | 8 |
| 4 | 42 (42) | m (m) | r (r) | 14 (13) | 6 |
| 5 | 18 (19) | m (m) | r (l) | 8 (9) | 0 |
| 6 | 22 (21) | m (m) | r (r) | 16 (15*) | 0 |
| 7 | 18 (20) | m (m) | r (r) | 9* (10) | 8 |
| 8 | 24 (24) | m (m) | r (r) | 15* (17) | 0 |
| 9 | 38 (38) | m (m) | r (r) | 17 (16) | 4 |
| 10 | 20 (19) | m (m) | r (r) | 9 (10) | 4 |
| 11 | 62 (60) | m (m) | r (r) | 14 (15) | 0 |
| 12 | 31 (33) | m (m) | r (r) | 13 (11) | 0 |
| 13 | 40 (40) | m (m) | r (r) | 14 (16) | 0 |
| 14 | 21 (19) | m (m) | l (r) | 13* (12*) | 0 |

on each page. The examiner asks the subject to make as many different figures as possible in one minute per sheet. Performance is scored for the number of unique patterns and for the number of perseverations (repetitions).

### *3.3. Executive functions: Planning and task management*

#### *3.3.1. Wisconsin card sorting test* (*WCST*) *[27]*

This instrument assesses "supervisory attentional control", "abstract behaviour" and "ability to react flexibly to changes in feedback". The subject is given a pack of 64 cards on which are printed one to four instances of four different symbols (triangle, star, cross, or circle) in red, green, yellow, or blue. The subject's task is to place the cards one by one under four stimulus cards, according to a principle that the patient must deduce from the pattern of the examiner's response to the subject's placement of cards. Scores used are the number of categories achieved, errors and perseverative errors.

#### *3.3.2. Working memory* (*subtest of the CBAA*) *[43]*

This test is designed as a typical two-back paradigm. A sequence of one-digit numbers is presented on the computer screen at a rate of approximately 0.5 per second and subjects have to indicate repetitions occurring after one intervening item. A total of 200 stimuli including 30 repetitions are delivered. The ability to maintain and actualise information is assessed. In this working memory paradigm RTs, errors and omissions were scored.

### *3.4. Different attention processes*

#### *3.4.1. "d2 Concentration-Endurance-Test" [7]*

The "d2 test" is letter cancellation task, which requires visual selective and sustained attention. It consists of rows of the letters "d" and "p". The patient is instructed to cross out all letters "d" having two small dashes. Letters "d" with no dash, one, three or four dashes as well as all different versions of the letter "p" are to be ignored. The performance is scored for errors and number of targets crossed out within the allotted time.

#### *3.4.2. Alertness* (*Subtest of the CBAA*) *[43]*

Subjects are instructed to react whenever an oblique cross (3.6 degrees visual angle) appears on the screen. In one condition (defined as phasic alertness), there are 40 visual stimuli each preceded by an auditory warning stimulus (interval between warning and imperative stimulus 300 to 700 ms), while in the second condition no warning tone is employed. To account for the effects of fatigue an ABBA-design was chosen. Reaction time is the most important measure in this test.

#### *3.4.3. Divided attention* (*Subtest of the CBAA*) *[34]*

This test assesses the ability to allocate attentional resources in parallel to two different tasks in two different modalities. A visual display containing a dot matrix and a concurrent series of auditory tones are presented. With an intertrial interval of 3000 ms crosses exchange some positions of the dot matrix with the subjects' task being to respond whenever this arrangement of crosses yields a square shape. At the same time subjects have to scan the series of auditory tones and have to press a button whenever a tone is repeated. 100 visual and 200 auditory stimuli are presented. In this paradigm RTs, errors and omissions were scored.

*3.4.4. Vigilance* (*Subtest of the CBAA*) *[43]*

This test assesses the ability to attend to visual stimuli over a longer period of time (30 minutes). Subjects have to attend to a bar that is moving up and down. Their task is to answer movements of larger amplitude by pressing a button. RTs, errors and omissions were computed separately for successive 5-minute periods.

### *3.5. Other neuropsychological domains*

*3.5.1. Verbal Learning Test* (*VLT*) */ Non-verbal Learning Test* (*NVLT*) *[41]*

These tests are designed like the Recurring Figures Test (RFT) to assess figural and verbal recognition. In these tests the stimulus material consists of 20 cards on which are drawn geometric or irregular nonsense figures (NVLT) or regular non-words (VLT). After looking at each of these cards in succession, the subject is shown a pack of 140 cards one by one for three seconds each. The subject must indicate which of the cards had been shown previously. False positive responses are subtracted from correct responses to correct for guessing.

*3.5.2. Finger tapping Test* (*FTT*) *[34]*

This test examines motor performance. Subjects have to press a tapping key for a 10–second period with the dominant and non-dominant hand. This procedure is repeated 5 times and an average score for each hand is obtained. The number of taps is analysed.

*3.5.3. Statistical analysis*

Analysis was performed in two steps. First, results from the entire test-battery were entered into a multivariate analysis of variance (MANOVA). This was done to assess whether there are overall differences in the neuropsychological status of Tourette patients and controls. However, since this procedure does not allow examining the neuropsychological profile in detail, this was followed by a Mann-Whitney-U-test, which was done separately for each test. This latter step was viewed in the sense of a descriptive statistic as proposed by Abt [1].

Furthermore we made a comparison of patients with primozide treatment and patients without. Test data were entered into an analysis of variance (ANOVA).

## 4. Results

The MANOVA statistic showed a significant difference between patients and controls (F (19,26) = 39.3, $p < 0.001$). The results are detailed in Tables 2 and 3. No influence of drug therapy was found for any of these neuropsychological tests neither in reaction time, in errors nor in omissions

While TS/OCD patient's test performance was within the normal range for most of the tests, they performed significantly worse than the control subjects in many tests measuring monitoring and response inhibition: in the Colour-Word-Interference Test with respect to errors; they were slower in the CBAA subtest Response flexibility and in the CBAA subtest Intermodal comparison. The results of the fluency tests, the Controlled Oral Word Association Test and the Ruff Figural Fluency Test, and those measuring task management and planning, the CBAA subtest Working memory with respect to errors and the WCST (except with respect to omissions), did not differ between groups.

Most of the tests from other psychological domains like attention and memory tests, the reaction time of the Number Connection Test, the CBAA subtest Alertness, the NVLT, and VLT did also not differ between groups. Exceptions were found only with respect to errors: Patients had more incorrect answers (false positives) in the "D2" test ($p < 0.004$), but also fewer errors than controls. In fact, they worked slower, but with more accuracy (correct answers – errors did not differ significantly between these groups). Additionally they made more errors in the vigilance task (CBAA) and the alertness task (CBAA). In theses cases one could argue, that the reduced performance in these tests is based on a disturbed error monitoring function not an attentional dysfunction. Moreover their motor performance measured by the finger tapping speed of the dominant hand was significantly faster than that of control subjects.

## 5. Discussion

In line with previous findings [20,24] TS/OCD patients had the same duration of education as controls, no deficits on memory tasks and none of any type of attention task (Table 4). The available body of scientific evidence suggests that persons with TS have normally distributed intellectual ability [9]. No effect of drug treatment was found in any of these neuropsychological test parameters. In addition, TS/OCD patients were not disturbed in executive functions in general.

Table 2
Means of median RTs, means of raw scores and results of the Mann-Whitney-U-test of the executive function subcomponents

| Test | | TP Mean (SD) | Controls Mean (SD) | Signif. |
|---|---|---|---|---|
| *Monitoring & response inhibition* | | | | |
| CWIT | RT (s) | 23.5 (4.5) | 22.1 (6.5) | $p = 0.51$ |
| CWIT | errors | 6.5 (1.7) | 2.0 (1.8) | $p < 0.01^*$ |
| Response inhibition (CBAA) | RT (ms) | 572 (94.4) | 563 (95.0) | $p = 0.31$ |
| Response inhibition (CBAA) | errors | 1.1 (1.5) | 0.7 (1.1) | $p = 0.42$ |
| Response inhibition (CBAA) | omissions | 0.1 (0.3) | 0.05 (0.3) | $p = 0.66$ |
| Response flexibility (CBAA) | RT (ms) | 925 (283) | 744 (158) | $p < 0.05^*$ |
| Response flexibility (CBAA) | errors | 9.3 (11.0) | 5.3 (5.8) | $p = 0.23$ |
| Intermodal comp. (CBAA) | RT (ms) | 484 (122) | 389 (75) | $p < 0.05^*$ |
| Intermodal comp. (CBAA) | errors | 0.8 (1.3) | 0.5 (0.7) | $P = 0.45$ |
| Intermodal comp. (CBAA) | omissions | 0.5 (0.8) | 0.2 (0.3) | $P = 0.20$ |
| *Productivity & fluency* | | | | |
| COWAT No. words | | 32.7 (10.0) | 35.7 (10.9) | $p = 0.45$ |
| RFFT unique designs | | 98.2 (14.7) | 103.4 (25.7) | $p = 0.51$ |
| RFFT perseverations ratio | | 0.74 (0.39) | 0.62 (0.28) | $p = 0.35$ |
| *Planning & task management* | | | | |
| WCST | No. categ. | 5.1 (1.6) | 5.6 (0.8) | $p = 0.31$ |
| WCST | No. pers. err. | 0.9 (1.5) | 0.7 (0.9) | $p = 0.67$ |
| Working Memory (CBAA) | RT (ms) | 745 (175) | 666 (210) | $p = 0.28$ |
| Working Memory (CBAA) | errors | 6.6 (7.7) | 6.9 (5.5) | $p = 0.90$ |
| Working Memory (CBAA) | omissions | 3.5 (3.1) | 1.1 (0.8) | $p < 0.01^*$ |

*Refers to significant differences.

Table 3
Means of median RTs, means of raw scores and results of the Mann-Whitney-U-test of the attention tests

| Test | | TP | Controls | Sig. Mean (SD) | Mean (SD) |
|---|---|---|---|---|---|
| Simple reaction time (CBAA) | RT (ms) | 234 (51.6) | 215 (45.4) | | $p = 0.31$ |
| Simple reaction time (CBAA) | errors | 1.1 (1.4) | 1.1 (1.0) | | $p = 0.99$ |
| Phasic Alertness (CBAA) | RT (ms) | 227 (53.7) | 221 (46.2) | | $p = 0.75$ |
| Phasic Alertness (CBAA) | errors | 2.0 (1.0) | 1.1 (1.3) | | $p < 0.02^*$ |
| Divided Attention (CBAA) | RT (ms) | 709 (54.9) | 708 (50.5) | | $p = 0.96$ |
| Divided Attention (CBAA) | errors | 1.8 (1.8) | 1.2 (2.1) | | $p = 0.42$ |
| Divided Attention (CBAA) | omissions | 1.6 (1.4) | 1.3 (0.6) | | $p = 0.46$ |
| Vigilance (CBAA) | RT (ms) | 497 (86) | 473 (85) | | $p = 0.46$ |
| Vigilance (CBAA) | errors | 17.9 (12.0) | 7.1 (11) | | $p < 0.02^*$ |
| Number connection test(NCT) | RT (ms) | 77.5 (17.3) | 77.5 (26.1) | | $p = 0.99$ |
| D2 | No. correct | 353.2 (88.5) | 409.3 (51.3) | | $p < 0.04^*$ |
| D2 | No. errors | 16.7(8.7) | 27.7(28.3) | | $p = 0.17$ |
| FTT dominant hand | No. taps | 57.1 (10.3) | 52.5 (4.5) | | $p < 0.04^*$ |
| FTT non dominant hand | No. taps | 51.6 (8.2) | 48.3 (3.2) | | $p = 0.34$ |

*Refers to significant differences.

A significant impairment was found in only one subcomponent of executive functions: monitoring and response inhibition. TS/OCD patients made significantly more errors in CWIT, Vigilance (CBAA) and Alertness (CBAA). Furthermore they made more errors in Response flexibility (CBAA) and Intermodal comparison (CBAA) (n.s.). In the Intermodal comparison (CBAA) and Response flexibility (CBAA) they showed significantly slowed reaction times. The increased number of errors in the two attention tasks (Vigilanzss and Alertness) cloud be based on a disturbed error monitoring function and not an original attentional dysfunction. By contrast, the other two components of executive function "cognitive productivity and fluency" and "task management and planning" were undisturbed (except the omissions in the working memory task) at least with respect to the current test battery. The intact fluency function of our sample is in line with the results of Mahone et al. [23], who found a normal performance on fluency tasks in TS children. Here one could reason

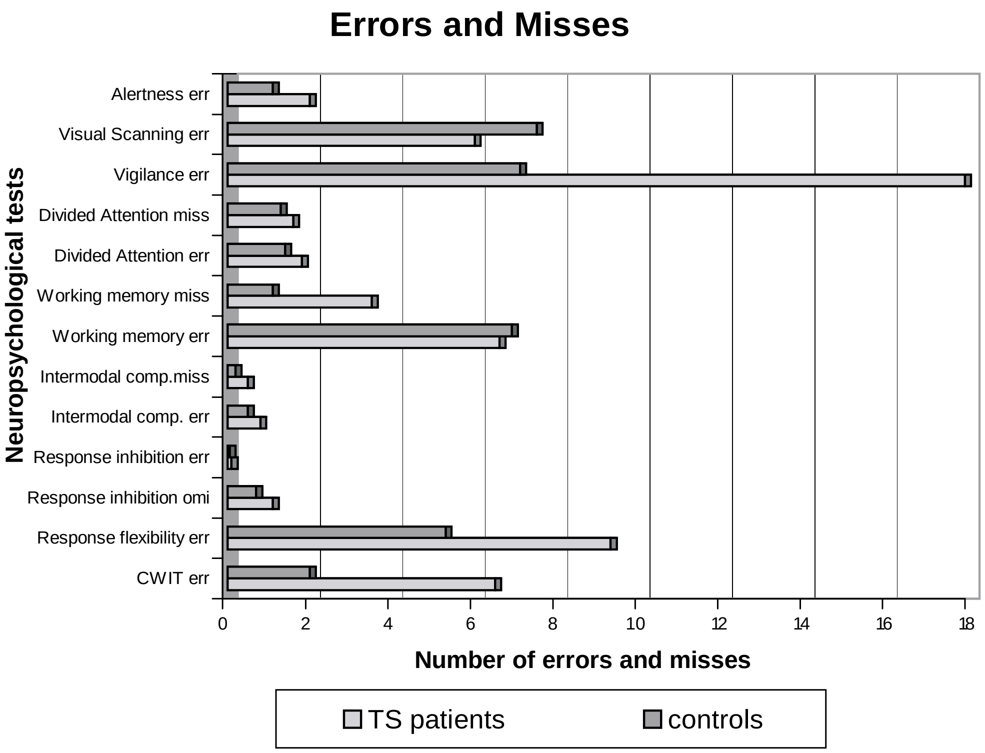

Fig. 1. Errors and omissions in neuropsychological tests for TS patients and controls.

again that the omissions in the working memory task (CBAA) are based more on error monitoring than on task mangement and planning.

The current results are in line with several other studies suggesting that the TS and / or OCD can lead to deficits in executive functions and abnormal functioning of the frontal lobe [10]. Likewise, Gedye [14] interpreted neuropsychological as well as motor and vocalization symptoms of TS as possible signs of a deficit in frontal executive functions. Such interpretations are supported by functional imaging results. For example, George et al. [15] showed an increase in frontal cerebral blood flow using the Single Photon Emission Computed Tomography (SPECT) technique, whereas the study of Braun et al. [6] showed a decreased glucose utilization rate in the frontal brain region with the Positron Emission Tomography (PET) technique. These findings have fuelled speculations about disturbed basal ganglia-frontal pathways in TS and abnormal discharges in the frontal lobes as the final common dysfunction in the Tourette Syndrome [14].

With respect to error processing, there have been a number of studies that have linked this executive function to the frontal lobe, in particular to the anterior part of the cingulate gyrus and the lateral frontal cortex. In the electrophysiological domain, an event-related brain potential component associated with errors (error related negativity) has been localized to the anterior cingulate gyrus [8] with possible contributions also from lateral frontal cortex [32]. Interestingly, this component has been found to be of enhanced amplitude in both TS [21] and OCD [16]. Additional evidence regarding the functional anatomy of the human error processing system has come from functional magnetic resonance imaging studies [8,22] that again pinpointed the anterior cingulate gyrus and lateral frontal cortex.

Greenberg [18] has summarized neuroanatomical and functional imaging studies in TS and OCD with the conclusion that frontal-striatal dysfunction is underlying many clinical aspects of both disorders. We agree with this notion but, more specifically, we propose that executive disturbances specifically impaired error processing and self-monitoring, might be the consequence of dysfunction of fronto-striatal loops. The basic pathomechanism may be a hyperactive frontal-striatal-thalamic-frontal circuit as has been hypothesized by others [18]. Error processing was associated with bilateral activity in the anterior cingulate, the dorsolateral prefrontal and left prefrontal cortex [32]. The anterior cingulate has an important function in the pro-

Table 4
Means of raw scores and results of the Mann-Whitney-U-test of the memory tests

| Memory Tests | | TP Mean (SD) | Controls Mean (SD) | Sig. |
|---|---|---|---|---|
| NVLT geom. | Score | 15.3 (3.3) | 15.8 (2.7) | $p = 0.66$ |
| NVLT non geo. | Score | 4.3 (4.5) | 5.3 (4.3) | $p = 0.55$ |
| VLT | Score | 36.6 (13.6) | 33.2 (10.9) | $p = 0.47$ |

cessing of emotion and forms a functional network with other regions such as the prefrontal cortex related to inhibitory processes [35].

The heterogeneous nature of the aetiology of TS/OCD must be stressed here as a likely reason for the heterogeneity of neuropsychological results. Several caveats about executive functions have to be kept in mind. The executive functions are not one process, but different sub-processes, which can be affected independently [25,40]. For more clarity, a subdivision in 3 subcomponents is useful [26]. Apparently, we found normal performance of TS/OCD in tests measuring fluency functions including the COWAT and RFFT [23], and in those quantifying planning and task management like the Subtest Working memory (CBAA) and WCST. In contrast, tests measuring inhibition of incorrect responses, selective attention to the relevant stimulus and set shifting were not fulfilled successfully. TS/OCD patients made more errors in CWIT (Fig. 1), and they perform significantly slower in the CBAA subtest "Response flexibility" and in the CBAA subtest "Intermodal comparison". In general we found a predominantly high rate of errors in different subgroups of executive tests, which leads us to the conclusion that one of the main cognitive disturbances in TS/OCD is error detection and response inhibition. Additionally, TS/OCD patients made more errors in the vigilance and phasic alertness task, which requires also a monitoring function over time. This may be due to the fact that the construct of executive functions encompasses a number of sub-functions not all of which were measured by different tests of executive functions.

In conclusion, we found evidence for a disturbance of a subcomponent of executive functions, namely the competence for self-monitoring, error detection and response inhibition mediated by the frontal lobes in our sample of TS/OCD patients. No specific impairment of other cognitive functions such as memory or attention was revealed. Only the disturbed error monitoring was mirrored by these functions. This is supported by the clinical experience that TS/OCD patients can achieve superior professional standing and exhibit normal intellectual functions. At the same time our results underscore the importance of further investigations of executive functions and their subcomponents for the understanding of the Tourette Syndrome.

## Acknowledgement

Supported by grants from the DFG Mu 1311/3-2, Mu 1311/7-1 and Mu 1311/6-1.
